# Different concentrations of C5a affect human dental pulp mesenchymal stem cells differentiation

**DOI:** 10.1186/s12903-021-01833-4

**Published:** 2021-09-24

**Authors:** Jie Liu, Xiaoling Wei, Junlong Hu, Xiaohan Tan, Xiaocui Kang, Li Gao, Ning Li, Xin Shi, Mengtong Yuan, Weiping Hu, Mingyue Liu

**Affiliations:** 1 Department of Radiology, The Second Affiliated Hospital of Harbin Medical University, Harbin, 150086 Heilongjiang People’s Republic of China; 2 Department of Prosthodontics, The Second Affiliated Hospital of Harbin Medical University and The Key Laboratory of Myocardial Ischemia Ministry of Education, Harbin, 150086 Heilongjiang People’s Republic of China; 3 Plastic Surgery Hospital of Chinese Academy of Medical Sciences and Peking Union Medical College, Beijing, People’s Republic of China; 4 Department of Prosthodontics, The Second Affiliated Hospital of Harbin Medical University, Harbin, 150086 Heilongjiang People’s Republic of China; 5 Department of Oral and Maxillofacial Surgery, The Second Affiliated Hospital of Harbin Medical University, Harbin, 150086 Heilongjiang People’s Republic of China; 6 Department of Cardiology, The Second Affiliated Hospital of Harbin Medical University and The Key Laboratory of Myocardial Ischemia Ministry of Education, Harbin, 150086 Heilongjiang People’s Republic of China; 7 Department of Prosthodontics, The Second Affiliated Hospital of Harbin Medical University, No. 246, Xuefu Road, Harbin, 150086 Heilongjiang People’s Republic of China; 8 Department of Prosthodontics, The Second Affiliated Hospital of Harbin Medical University and The Key Laboratory of Myocardial Ischemia Ministry of Education, No. 246, Xuefu Road, Harbin, 150086 Heilongjiang People’s Republic of China

**Keywords:** C5a, DPSCs, DSP, odontoblast, Differentiation

## Abstract

**Background:**

During the process of deep decay, when decay approaches the pulp, an immune response is triggered inside the pulp, which activates the complement cascade. The effect of complement component 5a (C5a) on the differentiation of dental pulp mesenchymal stem cells (DPSCs) is related to dentin reparation. The aim of the present study was to stimulate DPSCs with different concentrations of C5a and evaluate the differentiation of odontoblasts using dentin sialoprotein (DSP).

**Methods:**

DPSCs were divided into the following six groups: (i) Control; (ii) DPSCs treated with 50 ng/ml C5a; (iii) DPSCs treated with 100 ng/ml C5a; (iv) DPSCs treated with 200 ng/ml C5a; (v) DPSCs treated with 300 ng/ml C5a; and (vi) DPSCs treated with 400 ng/ml C5a. Flow cytometry and multilineage differentiation potential were used to identify DPSCs. Mineralization induction, Real-time PCR and Western blot were conducted to evaluate the differentiation of odontoblast in the 6 groups.

**Result:**

DPSCs can express mesenchymal stem cell markers, including CD105, CD90, CD73 and, a less common marker, mesenchymal stromal cell antigen-1. In addition, DPSCs can differentiate into adipocytes, neurocytes, chondrocytes and odontoblasts. All six groups formed mineralized nodules after 28 days of culture. Reverse transcription-quantitative PCR and western blotting indicated that the high concentration C5a groups expressed higher DSP levels and promoted DPSC differentiation, whereas the low concentration C5a groups displayed an inhibitory effect.

**Conclusion:**

In this study, the increasing concentration of C5a, which accompanies the immune process in the dental pulp, has demonstrated an enhancing effect on odontoblast differentiation at higher C5a concentrations in vitro.

## Background

Caries is caused by bacteria that chronically destroy the hard tissues of the teeth. When deep decay approaches the pulp, bacterial metabolites stimulate the pulp via dentinal tubules, causing irritation of the pulp, even when the pulp is not exposed. The clinical treatment of deep decay often comes with uncertainty and usually fails. During the early stages of stimulation, immune cells in the pulp that reach the damaged site release various inflammatory factors to attract more immune cells, resulting in cascade amplification. However, the pulp is located in a relatively closed cavity, which makes the cycle of metabolism weaker; therefore, dead cells enhance the amplification of the inflammatory signal, and eventually, pulpitis occurs. A treatment strategy to control the pulpal immune response, induce repair and avoid the occurrence of pulpitis has not been identified.

Deep decay that approaches the pulp initially induces an immune response, leading to complement activation. Each complement component (C2-C5) is cleaved into two or more fragments, which are represented by a and b, for example. C2a, C3b, C4b and C5b bind directly or indirectly to the target cell membrane and participate in the cytolytic function of complement, while C3a and C5a are released into the fluid phase. C5a is derived from the α-polypeptide via C5-convertase-mediated cleavage and displays an important role during inflammation. Furthermore, C5a is an anaphylatoxin that possesses potent chemotactic activity. Among the anaphylatoxins, C5a displays the strongest effect, with an effect 20 and 2,500 times stronger than C3a and C4a, respectively; therefore, C5a was chosen for the present study. Studies conducted over the past several years have revealed that C5a is a strong chemoattractant that can activate DPSCs migrating to the damaged tissue to initiate the process of dentin-pulp regeneration [1–3]. Any repair of local tissue can lead directly to the regeneration of dentin-pulp. Previous studies have also displayed that during the early stage of dentin-pulp regeneration, C5a displays an important role. Moreover, pulp progenitor cells are selectively recruited via the C5a gradient [3]. During the inflammatory response, the binding of C5a to the C5a receptor (C5aR) can mediate the development of inflammation, by inducing increased intracellular calcium levels and intracellular signaling cascades, such as the recruitment and activation of inflammatory cells. The release of granzymes and histamine increases the expression of cell adhesion molecules. Collectively, the aforementioned processes enhance the immune response. The relationship between C5a and regeneration has been reported in liver [4], cardiac [5] and bone [6] tissues. The liver of C5-deficient mice can regenerate following an injection of C5 or C5a [7], and in cardiac tissue, C5a induced the migration and differentiation of progenitor cells [5]. Moreover, following bone fracture, the expression of C5aR increased significantly during the process of osteogenic differentiation of mesenchymal progenitors. C5a also induced the migration of mesenchymal cells and osteoblasts during the regeneration progress, indicating that C5a was involved during the regeneration of fractured bone [6].

DPSCs originate from the ectoderm and as adult progenitor cells they display mesenchymal features [8, 9], including clonality and self-renewal capacity [10, 11]. Although the exact marker of DPSCs has not been previously identified, dental pulp mesenchymal stem cell populations have been widely characterized by the expression of STRO-1, which is a bone marrow cell membrane antigen [12, 13]. However, other markers, including the melanoma cell adhesion molecule/CD146 [a marker of perivascular mesenchymal stem cells (MSCs)] [14–16], the low-affinity nerve growth factor receptor (CD271) [15, 17, 18], the mesenchymal stem cell antigen (MSCA-1/tissue non-specific alkaline phosphatase) [19] and the neural cell adhesion molecule (CD56; a marker of neural and muscular MSC populations) [15, 19, 20], have been identified. A recent study reported that a series of mesenchymal stromal/stem cell markers were expressed in DPSCs, including CD56, CD146, CD271, MSCA-1 and STRO-1 [21]. The CD31 negative dental pulp cells population contained ~ 1.4% CD56^+^ cells, 1.5% CD146^+^ cells, 2.4% CD271^+^ cells and 6.3% MSCA-1^+^, indicating the exact expression of mesenchymal stromal/stem cell markers. Furthermore, there were few STRO-1^+^ cells (< 0.3%) compared with others; therefore, the detection of STRO-1 expression was not optimal compared with previous studies. Mesenchymal stem cells were detected by flow cytometry via the expression of CD34, CD45, CD73, CD90, CD105 and MSCA-1.

Dentin sialophosphoprotein (DSPP) is a non-collagenous matrix protein of odontoblasts, which can be cleaved to dentin phosphoprotein and dentin sialoprotein (DSP) [22]. DSP has been reported to be involved during the process of dentin mineralization, and its essential role during dentinogenesis has been confirmed [23]. Therefore, a number of studies have used DSP and DSPP as odontoblasts differentiation markers. DPSC differentiation has been optimized using 5 mM β-glycerophosphate, which induced a higher expression of DSP [24]. In a previous study, DSP was used as a marker for odontoblasts differentiation [25].

When deep decay occurs, the decay is removed and further external stimuli are prevented. At this time, aseptic inflammation in the pulp is the key issue that prevents the successful self-healing of the pulp. Regeneration of the pulp requires a certain degree of the immune response, growth factors and signal molecules to establish an environment conducive to the formation of dentin, which are particularly important for the rapid formation of reparative dentin. Recently, it was reported that 1 μg/ml lipoteichoic acid (LTA) and lipopolysaccharide (LPS) can stimulate dental pulp cells to release C5a and interleukin-6 [26].

In addition to promoting the migration of stem cells, whether C5a displays a positive role during tooth regeneration has not been previously reported. After stem cells migrate, whether C5a plays an important role during the next stage of regeneration, for example by promoting the differentiation of odontoblast cells, is not completely understood. Therefore, the aim of the present study was to analyze the effect of different concentrations of C5a on DPSC differentiation in order to investigate whether C5a displayed an effect on accelerating the differentiation of odontoblasts to accelerate the formation of reparative dentin.

## Methods

### Primary cell culture

Human dental pulp was collected from immature third molars, and the 1/3 tip of the pulp was cut off and subsequently, the explant outgrowth method was performed [24]. For each experiment, three different donors were used (n = 12; 3–4 molars per donor; the male to female ratio of cases was 1:1, age18-25 years,the range of date was from Jan. 2019 to March 2019). Exodontia was performed by the Department of Oral and Maxillofacial Surgery in the Second Affiliated Hospital of Harbin Medical University. The present study was approved by the Institutional Ethics Committee of the Second Affiliated Hospital of Harbin Medical University. Written informed consent was obtained from all donors. All methods were performed in accordance with the relevant guidelines and regulations. The basic culture medium of DPSCs was Human Mesenchymal Stem Cell Growth Medium Kits (RASMX-90011, Cyagen Biosciences Guangzhou, China), including 10% FBS, 1% penicillin and streptomycin and 1% glutamine.

### Identification of DPSCs by CD34, CD45, CD73, CD90, CD105 and MSCA-1 using flow cytometry

Dental pulp cells were cultured in 75cm^2^ cell culture flasks (Corning, Inc.) and a total of 7 flasks were prepared. Cells were washed three times with PBS and digested using 2 ml pre-warmed 0.25% trypsin. Digestion was terminated and cells were counted. Cells were collected at a density of 1 × 10^6^ cells/ml into 15 ml centrifuge tubes. Cells were washed three times using PBS and centrifuged for 10 min at 300×*g* at room temperature. The supernatant was discarded and the cell pellets were incubated on ice for 30 min. Cells in one tube served as isotype-matched control. Cells in the remaining six tubes were incubated with monoclonal conjugated antibodies (Table 1) to identify pulp immunocompetent cells. Antibodies were used at a 1:10 dilution (5 μl antibody with 50 μl buffer). Subsequently, 1–2 ml buffer was added to each tube and centrifuged for 10 min at 300×*g* at room temperature to wash the cells. Cells were resuspended in 500 μl buffer. Flow cytometry was performed using the BD FACS Canto flow cytometer (BD Biosciences) and FACS Diva software (version 6.1.3) (BD Biosciences, San Jose, CA, USA).

**Table 1:** Monoclonal antibodies used in this study

Antibodies	Fluorochrome	Isotype	Manufacturer	Catalog
CD34	APC	Mouse IgG1	R&D SYSTEMS	FAB7227A-025
CD45	PE	Mouse IgG1	R&D SYSTEMS	FAB1430P-025
CD73	APC	Mouse IgG2B	R&D SYSTEMS	FAB5795A
CD90	PE	Mouse IgG2A	R&D SYSTEMS	FAB2067P
CD105	PE	Mouse IgG1	R&D SYSTEMS	FAB10971P-025
MSCA-1	PE	Mouse IgG1κ	Miltenyi Biotec	130-099-198

### Experimental groups

Cells were cultured in a 37 °C, 5% CO_2_ incubator and divided into six groups (n = 1 × 10^5^ cells/group, using Corning® 25cm^2^ Rectangular Canted Neck Cell Culture Flask with Vent Cap): (i) cells treated with basic cell culture medium containing 10 nmol/l dexamethasone, 5 mmol/l β-glycerophosphate and 50 mg/ml vitamin-C-phosphate as control; (ii) DPSCs treated with 50 ng/ml recombinant human complement component C5a protein (C5a; R&D Systems, Inc.); (iii) DPSCs treated with 100 ng/ml C5a; (iv) DPSCs treated with 200 ng/ml C5a; (v) DPSCs treated with 300 ng/ml C5a; and (vi) DPSCs treated with 400 ng/ml C5a. In addition to basic cell culture medium which included and C5a, 10 nmol/l dexamethasone (Sigma-Aldrich, USA), 5 mmol/l β-glycerophosphate (Sigma-Aldrich, USA) and 50 mg/ml vitamin-C-phosphate (Sigma-Aldrich, USA) were added to each tube to promote odontoblastic differentiation. The culture medium was replaced every other day with fresh culture medium containing the same concentration of C5a.

### Multilineage potential of DPSCs into adipocytes, odontoblasts chondroblasts and neurons in vitro

Cells at passage 3 were plated at a density of 1 × 10^5^ cells per well. Adipocyte differentiation was induced by culturing DPSCs with 0.5 μM isobutylmethylxanthine (Sigma-Aldrich, USA), 50 μM indomethacin (Sigma-Aldrich, USA) and 0.5 μM dexamethasone (Sigma-Aldrich, USA) in a 37 °C, 5% CO_2_ incubator for 3 weeks. Subsequently, cells were fixed in 4% paraformaldehyde for 30 min at room temperature and stained with a fresh Oil Red O solution for 1 h at room temperature. odontoblasts differentiation was induced by culturing DPSCs with 5 mM/l β-glycerophosphate, 10 nM/l dexamethasone and 50 mg/ml vitamin-C-phosphate in a 37 °C, 5% CO_2_ incubator for 3 weeks. Subsequently, cells were fixed using 95% ethanol for 30 min at 37 °C and stained with Alizarin Red S for 30 min at 37 °C. The cells for adipocyte and odontoblasts differentiation were observed under a light microscope (ZEISS, 37081 Goettingen, Germany)(original magnification, × 40). The chondrogenic cultures were fixed in 4% paraformaldehyde for 30 min at room temperature and stained with toluidine blue for 30 min in a 37 °C incubator. Neuronal differentiation of DPSCs was induced as previously described [27]. Briefly, cells were cultured with basic cell culture medium containing 500 μM β-mercaptoethanol (Sigma-Aldrich, USA) and 10 ng/mL βFGF (Sigma-Aldrich, USA) in a 37 °C, 5% CO_2_ incubator for 24 h. Subsequently, the culture medium was replaced with serum-free medium containing 2% DMSO and 100 μM butylated hydroxyanisole (Sigma-Aldrich, USA) at 37 °C, 5% CO_2_ incubator for 6 h. Cells were fixed in 4% paraformaldehyde at room temperature for 20 min. PBS-T (phosphate buffer saline with 0.1% Triton X-100) were used to permeabilize with at 4 °C for 10 min. Then we removed the PBS-T and washed the cells three times with PBS (phosphate buffer saline). Samples were treated with PBS-B (4% bovine serum albumin in PBS) at 37 °C for 30 min. Then we incubated the antibodies. The anti-Nestin antibody (1:200, SantaCruz, USA) was added into the samples at 4 °C overnight, then washed the samples with PBS for 5 min. The secondary antibody solution (1:200, EarthOx, California, USA) incubated the samples for 60 min at room temperature in the dark. Stained the samples with the nuclear dye 4,6-diamidino-2-phenylindole dihydrochloride (DAPI, Beyotime, CHN) for 5 min, washed with PBS 3 times and then fluorescent images were acquired. Immunofluorescence was detected using a fluorescence microscope (magnification, × 20; DMI14000B; Leica Microsystems GmbH, Wetzlar, Germany).

### Cytotoxicity (MTT) assay cultured by different concentrations of C5a

The effect of different concentrations of C5a on cell viability was assessed using an MTT assay. Cells were seeded (1 × 10^4^ cells/well) into 96-well plates. After 24 h, cells were not confluent, then cells were treated with 50, 100, 200, 300 or 400 ng/ml C5a in a 37 °C, 5% CO_2_ incubator for 24 h. The control group was incubated with normal medium. Subsequently, cell cytotoxicity was assessed using the Cell Proliferation Kit I (MTT; Roche Applied Sciences), according to the manufacturer’s protocol. Dimethyl sulfoxide (200 μl) was added to dissolve the formazan crystals. The absorbance of each well was measured at a wavelength of 490 nm using an ELISA reader (Thermo Fisher Scientific, Inc.). Cell viability was calculated according to the following formula: Cell proliferation rate (%) = [mean optical density (OD) of the group − mean of OD of zero-set group]/(mean OD of untreated group − mean of OD of zero-set group) × 100.

### Mineralization induction of all 6 groups

Cells at passage 3 were seeded (5 × 10^4^ cells/well) into 6-well plates, and cultured with different concentrations of C5a (50, 100, 200, 300 or 400 ng/ml) and mineralized medium (5 mM/l mll β-glycerophosphate, 10 nM/ml l dexamethasone and 50 mg/ml vitamin-C-phosphate). The control group was cultured in mineralized medium without C5a. Following culture in a 37 °C, 5% CO_2_ incubator for 28 days, cells were fixed using 95% ethanol for 30 min at 37 °C. Subsequently, cells were stained with 0.1% Alizarin Red S (Sigma-Aldrich; Merck KGaA) at 37 °C for 30 min to detect calcium accumulation. The mineralized nodules were observed under a light microscope (ZEISS, 37081 Goettingen, Germany)(original magnification, × 40). The quantification of matrix mineralization was conducted by cetylpyridinium ch loride, the alizarin red S stained cultures were incubated with 100 mM cetylpyridinium chloride for 1 h at 37 °C to solubilize and release calcium-bound alizarin red into solution. 200 ml aliquots were transferred to a 96-well plate and the OD570 nm of the solution was measured using a microplate reader (Shanghai Tanon Science Company). Mineralized nodule formation was represented as OD per μg of total cellular protein, determined by Bradford Protein assay. Experiments were performed in triplicate wells and repeated at least three times.

### Reverse transcription-quantitative PCR (RT-qPCR) of DSP and Actin expressions

Following culture for 28 days, total RNA was extracted from the cells using the RNeasy Mini kit used according to the manufacturer’s protocol (Qiagen GmbH). Total RNA was reverse transcribed into cDNA using the Transcriptor First Strand cDNA Synthesis kit used according to the manufacturer’s protocol (Roche Diagnostics GmbH). The following thermocycling conditions were used for reverse transcription: 50 °C for 60 min, 85 °C for 5 min and 4 °C for 10 min. Subsequently, qPCR was performed using FastStart Universal SYBR Green Master Rox Mix (Roche Diagnostics GmbH). The following thermocycling conditions were used for qPCR: 95 °C for 2 min; 40 cycles of 95 °C for 15 s and 60 °C for 30 s. The following primer pairs were used for qPCR: DSP forward, 5′-TTTCCGCTTGTCATCATCTCC-3′ and reverse, 5′-GGTGTCCTGGCACTACTGCAT-3′; and actin, forward 5′-GGGAAATCGTGCGTGACATT-3′ and reverse, 5′-GGAACCGCTCATTGCCAAT-3′. mRNA expression levels were quantified using the 2^−∆∆Cq^ method [28] and normalized to the internal reference gene actin.

### Western blotting of DSPP and Actin expression

Following culture for 28 days, total protein was extracted from the cells using cell lysis buffer, which consisted of 150 mM NaCl, 20 mM Tris (pH 7.5), 2.5 mM sodium pyrophosphate, 1% Triton X-100, 1% Na_3_CO_4_, 0.5 μg/ml leupeptin, 1 mM EDTA and 1 mM phenylmethanesulfonyl fluoride. Total protein was quantified using the Pierce™ BCA Protein Assay kit used according to the manufacturer’s protocol (Thermo Fisher Scientific, Inc.). Protein samples (20 μg/lane) were separated via 12% SDS-PAGE and transferred onto PVDF membranes by Trans-Blot SD Cell Semi-Dry Transfer (Bio-Rad Laboratories, Inc.). Subsequently, the membranes were blocked with 5% milk at room temperature for 1 h with gentle agitation. The membranes were incubated at 4 °C overnight with the following primary antibodies: Mouse anti-human DSPP (LFMB-21; cat. no sc-73632; 1:200; Santa Cruz Biotechnology, Inc.) and β-actin (cat. no. 20536-1-AP; 1:5,000; ProteinTech Group, Inc.). Following primary incubation, the membranes were incubated with the following secondary antibodies at room temperature for 1 h.: goat anti-mouse IgG (cat. no. SA00001-1; 1:10,000; ProteinTech Group, Inc.) and goat anti-rabbit IgG (cat. no. SA00001-2; 1:10,000; ProteinTech Group, Inc.). Protein bands were visualized using the Clarity MaxTM Western ECL substrate (Bio-Rad Laboratories, Inc.) and photographed using the Tanon 1000 digital image gel analytical system (Tanon Science & Technology Co., Ltd.). β-actin was used as the loading control. The bands were quantified using Image J (NIH, USA).

### Statistical analysis

One-way ANOVA followed by Bonferroni’s multiple comparisons was performed using SPSS software (version 13.0; SPSS, Inc.). P < 0.05 was considered to indicate a statistically significant difference. Data are expressed as the mean ± standard error of the mean.

## Results

### Identification of DPSCs by CD34, CD45, CD73, CD90, CD105 and MSCA-1 using flow cytometry

**Fig. 1:**
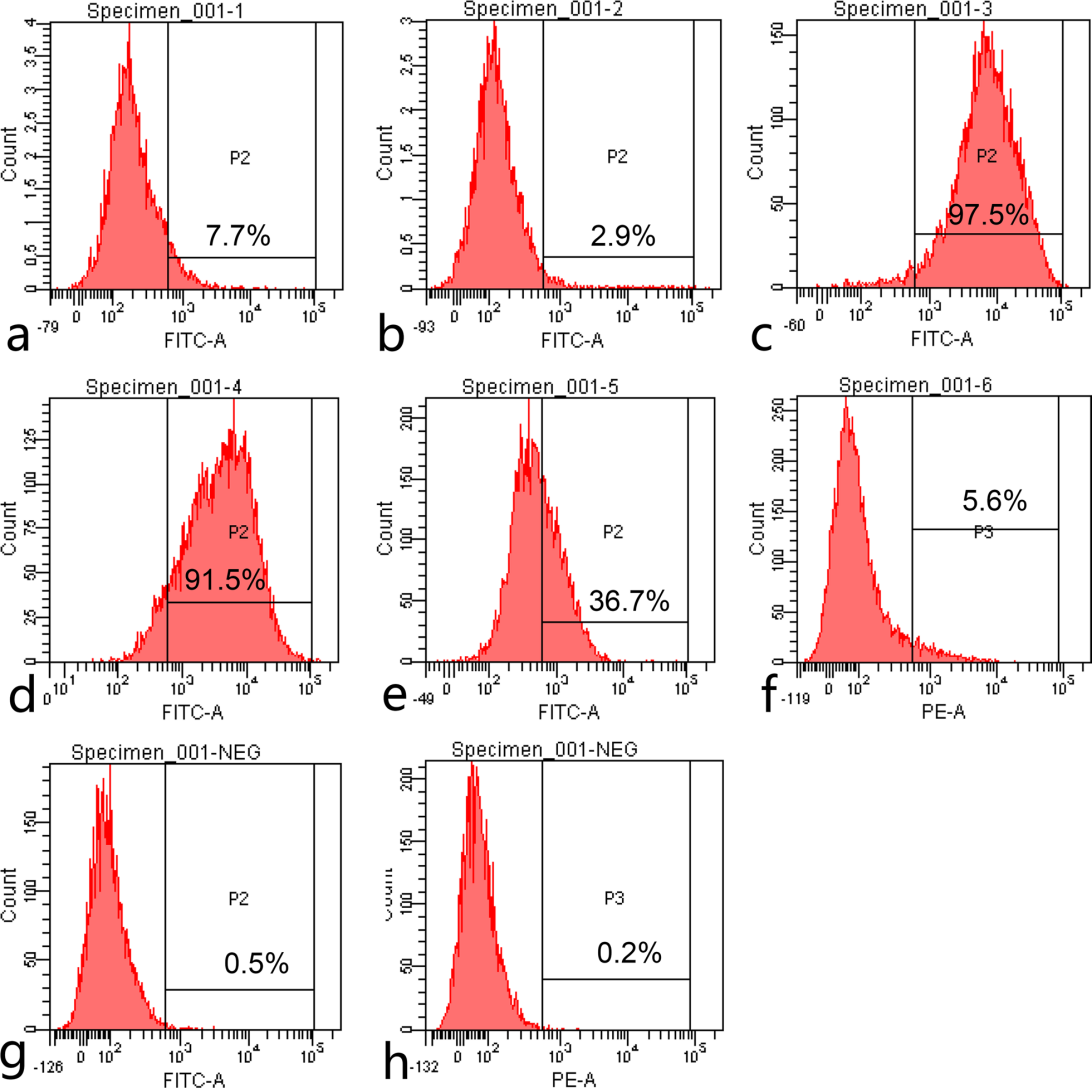
Expression profiles of stem cell surface markers in human dental pulp cells cultured with Mesenchymal Stem Cell Growth Medium, as determined by FACS flow cytometry. **A** CD34, **B** CD45, **C** CD73, **D** CD90, **E** CD105 and **F** MSCA-1. **G** and **H** showed the isotype controls. MSCA-1, mesenchymal stromal cell antigen-1. Levels of fluorescence greater than 1% compared to the isotype control signify positivity

Flow cytometry analysis indicated that CD34 (Fig. 1A), CD45 (Fig. 1B), CD73 (Fig. 1C), CD90 (Fig. 1D), CD105 (Fig. 1E) and MSCA-1 (Fig. 1F) were expressed by 7.7, 2.9, 97.5, 91.5, 36.7 and 5.6% of DPSCs, respectively. Levels of fluorescence greater than 1% compared to the isotype control signify positivity.

### Multilineage potential of DPSCs into adipocytes, odontoblasts and neurons

**Fig. 2:**
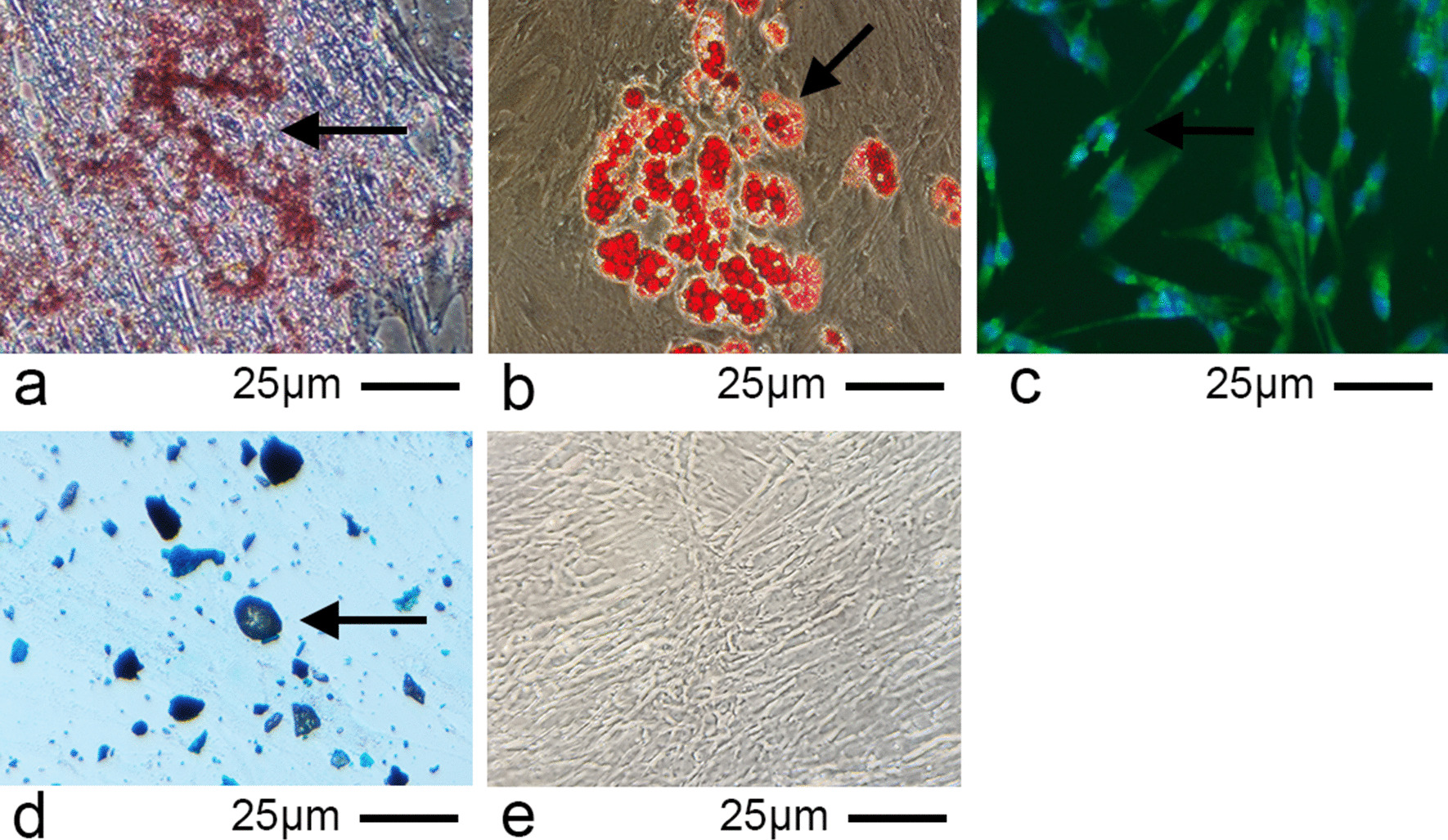
Multilineage differentiation capacity of dental pulp stem cells. **A** Arrows indicate odontoblasts differentiation, which was identified by the deposition of mineralized matrix, as determined by Alizarin Red S staining. **B** Adipogenic differentiation was indicated by the accumulation of neutral lipid vacuoles, as determined by Oil Red O staining. **C** Arrows indicate nerve cells, which were detected by immunocytochemical staining (magnification, × 40; scale bar, 25 m). **D** Chondroblasts were detected by toluidine blue staining. Arrows indicate representative stained cells. **E** Control cells (only growth medium)

The visible mineralized nodules are presented in Fig. 2A, which demonstrated the odontoblasts induction of DPSCs. The neutral lipid vacuoles presented in Fig. 2B indicated the adipogenic differentiation of the DPSCs. The neuronal differentiation of DPSCs was determined by Nestin immunocytochemical staining (Fig. 2C). Furthermore, the DPSCs were observed to differentiate into chondroblasts following induction (Fig. 2D).

### Cell morphology and proliferation of DPSCs cultured with different concentrations of C5a

**Fig. 3:**
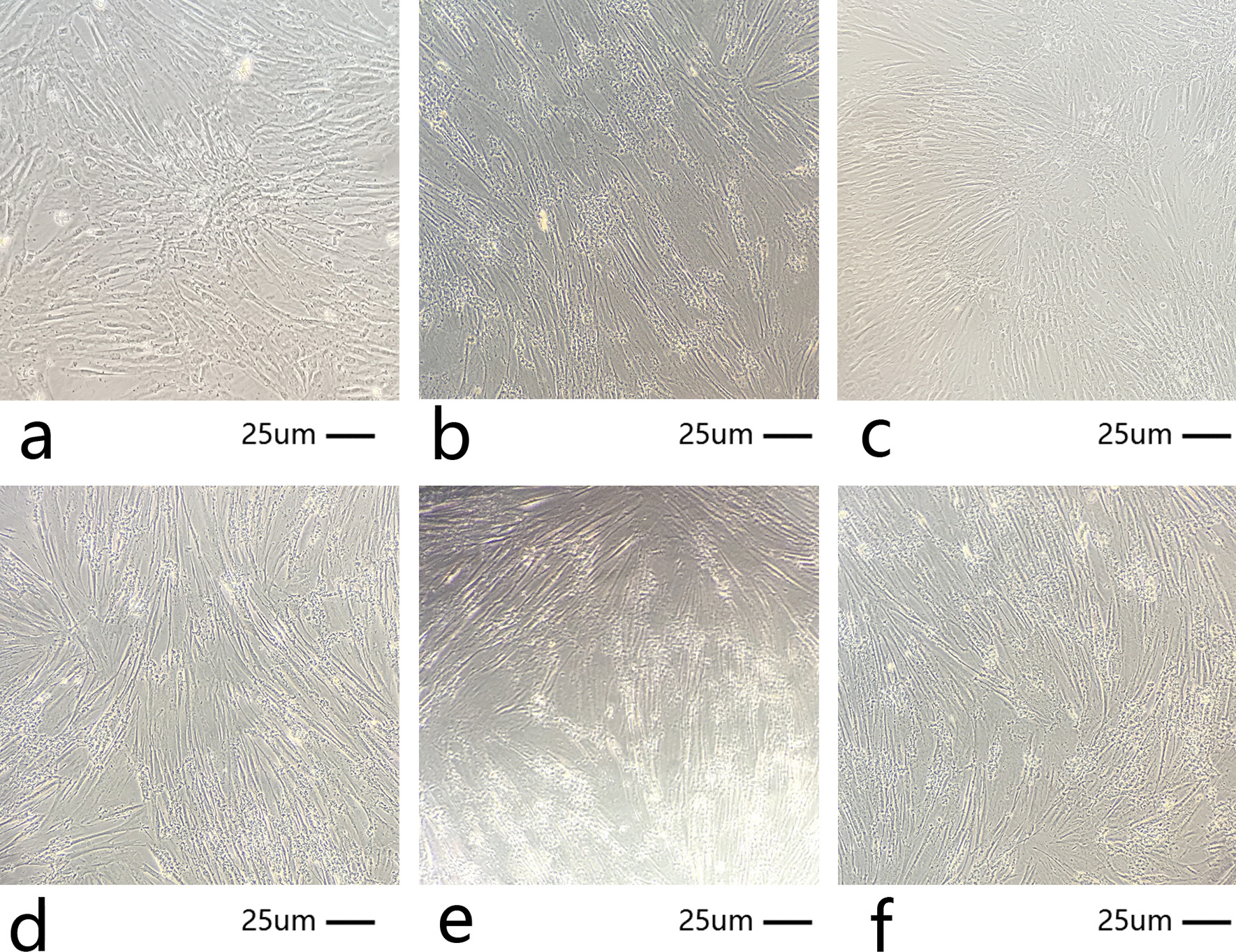
Morphological appearance of DPSCs cultured with different concentration of C5a for 2 weeks. **A** 50, **B** 100, **C** 200, **D** 300, **E** 400 ng/ml C5a groups and** F** Control group. DPSCs, dental pulp mesenchymal stem cells; C5a, complement component 5a

The cell morphology of cultured DPSCs incubated with different concentrations was assessed (Fig. 3). Cells in the six groups grew well with no differences compared with the control group. In all six groups, normal cell morphology was observed (Fig. 3).

**Fig. 4:**
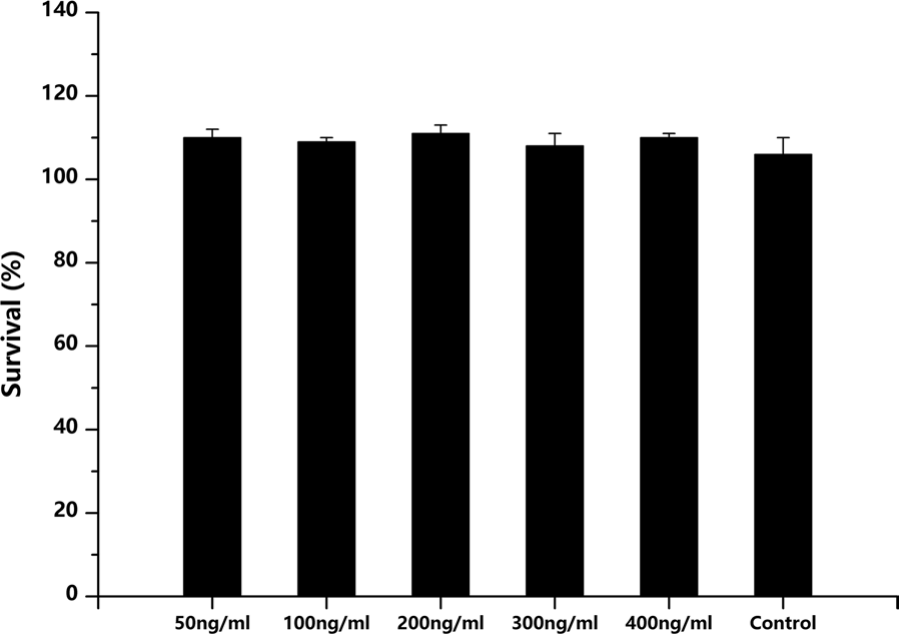
Cell viability was quantified using the MTT assay. Data are presented as the mean ± SEM

The results of the MTT assay indicated that there was no statistical significance among the experiment groups in Fig. 4, and no inhibitory effect on cell proliferation was observed in the six groups, which suggested that C5a did not influence DPSC proliferation. Cells incubated with 400 ng/ml C5a displayed a cell survival rate of 108.68 ± 12.35%. The results indicated that the five concentrations of C5a assessed in the present study displayed no toxic effect on the proliferation of DPSCs (Fig. 4).

### Odontoblast differentiation by RT-qPCR analysis and Western blot

**Fig. 5:**
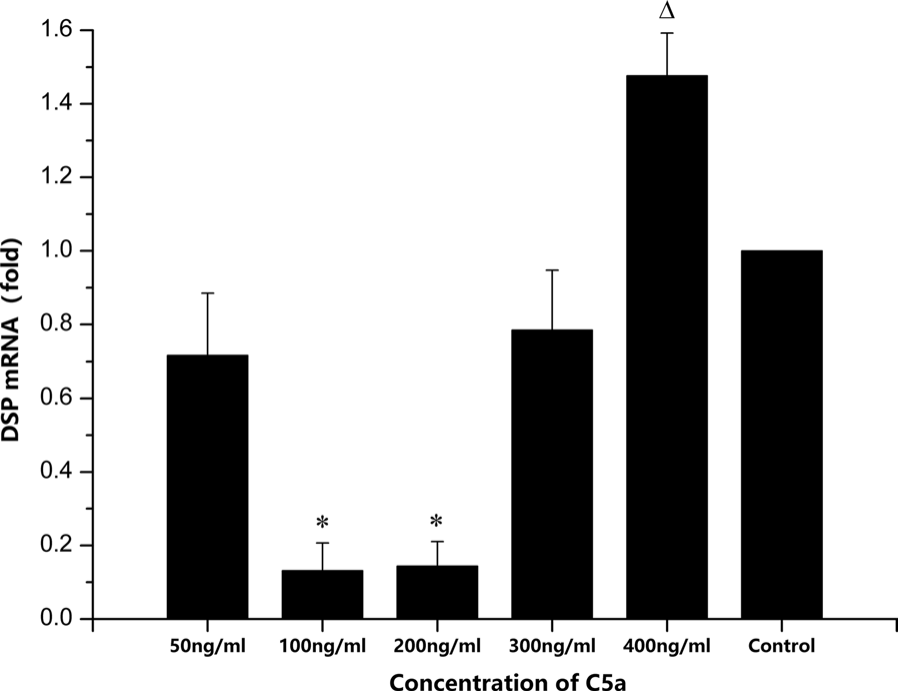
DSP mRNA expression in the different DPSC groups. Values were normalized to the internal reference gene actin. *P < 0.05, *showed 100 ng/ml and 200 ng/ml groups expressed significantly lower DSP mRNA compared with other groups. ^△^P < 0.05, ^△^showed 400 ng/ml group expressed significantly higher DSP mRNA. DSP, dentin sialoprotein; DPSC, dental pulp mesenchymal stem cell

**Fig. 6:**
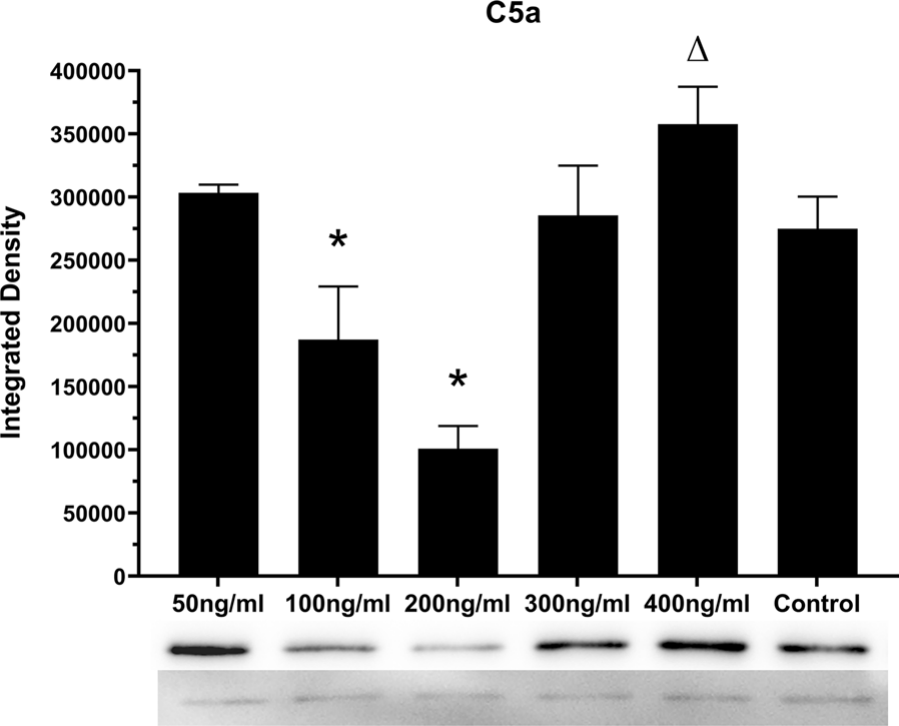
Effect of different concentration of C5a stimulation on DSPP protein expression in DPSC. All 6 groups were cultured in dentinogenic medium. DSPP expression was determined by Western blot after 28 days of culture time. Results are expressed as relative expression to Actin. Data are presented as mean ± SEM of 3 independent experiments. *P < 0.05, *showed 100 ng/ml and 200 ng/ml groups expressed significantly lower DSP protein compared with other groups.. ^**△**^P < 0.05, △showed 400 ng/ml group expressed significantly higher DSP protein. DSPP, dentin sialophosphoprotein; DPSC, dental pulp mesenchymal stem cell

Odontogenesis-associated gene DSP was expressed by all six groups, as determined by RT-qPCR (Fig. 5). However, DSP expression was weaker in groups ii and iii compared with the other three groups, whereas group v displayed notably higher gene expression. The western blotting results were similar to the RT-qPCR results. DSPP protein expression levels were weak in groups ii and iii, which were significantly lower compared with the other groups. Group v displayed the highest protein expression levels of DSPP (Fig. 6).

### Mineralization assay of all 6 groups

**Fig. 7:**
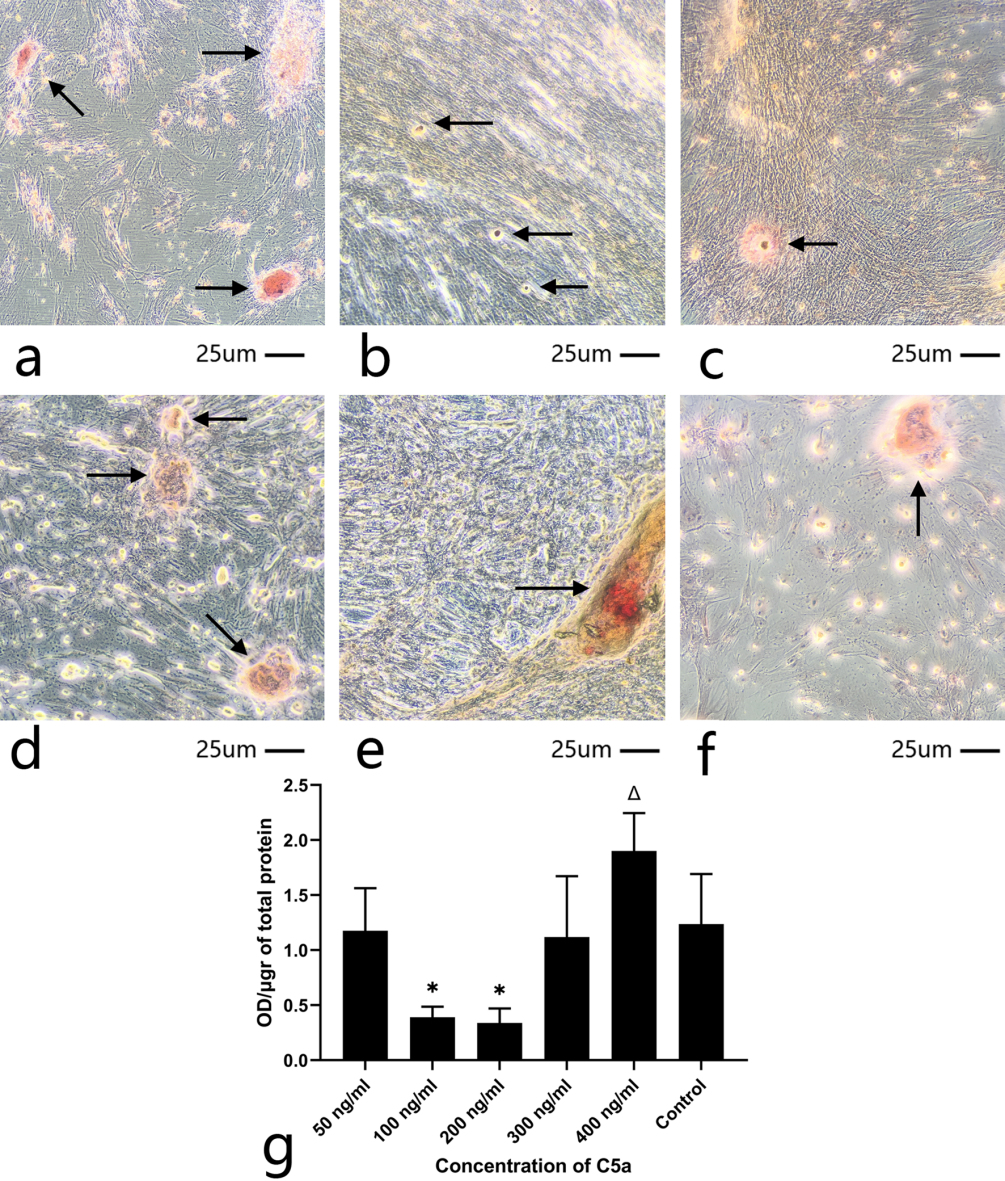
Cell morphology and mineralized nodules of hDPSCs cultured with C5a and mineralized medium for 28 days. **A** 50 ng/ml C5a culture hDPSCs (arrows nodules). **B** 100 ng/ml C5a culture hDPSCs displayed smaller and less mineralized nodules (arrows nodules). **C** 200 ng/ml C5a culture hDPSCs, displayed smaller and less mineralized nodules (arrows nodules). **D** 300 ng/ml C5a culture hDPSCs (arrows nodules). **E** 400 ng/ml C5a culture hDPSCs displayed bigger and more mineralized nodules (arrows nodules). **F** Control group. C5a, complement component 5a, hDPSCs, human dental pulp mesenchymal stem cell (arrows nodules). **G** Quantification of mineralized nodule formation. Data are shown as mean OD/µg of total protein ± SD (n = 6). *Indicates significant differences compared to conrol group (P < 0.05)

After 28 days of culture, mineralized nodules were observed in all six groups. Cells displayed an odontoblasts-like polygonal morphology with white mottled crystals. Red mineralized nodules were stained by Alizarin Red S staining and are presented in Fig. 7. Groups ii and iii displayed relatively smaller mineralized nodules compared with the other groups (Fig. 7B and C). A large mineralized nodule was observed in group v (Fig. 7E). Quantification of mineralized nodule formation was represented as OD per μg of total cellular protein (Fig. 7G). The data showed that group v had a prominent mineralized ability, while group ii and iii showed low degree of mineralization.

## Discussion

Caries and pulpitis are the most common diseases in the clinical diagnosis and treatment of the department of dentistry. Severe lesions will not only lead to the defect of tooth tissue, but also may cause damage to the surrounding tissues, such as periapical lesions and alveolar bone resorption, which will bring a lot of trouble for the follow-up treatment.Therefore, it is very important to control the development of the disease in the early stage, such as deep decay that approaches the pulp. In the case of deep caries that approaches the pulp, the complements in the pulp are activated first, then the complements summon all sorts of immune cells and signaling molecules for an immune response. During this process, the complement 5a express most during all complements, which is 20 times more than C3a and 2500 times more than C4a. Therefore, the immune response study in dental pulp on C5a is more meaningful.

The characteristics of DPSCs have been previously reported. Compared with other adult stem cells, such as bone marrow stem cells, DPSCs are superior, not only regarding regeneration and renovation properties, but also in other tissues and organs [29]. Odontoblast differentiation is the prerequisite to dental tissue engineering and dental restoration. A number of studies have promoted the differentiation of DPSCs into odontoblasts using a variety of factors, including bone morphogenetic protein 2 [30], neuropilin-1-FYN [30], cellular retinoic acid-binding protein 2 [31] and intraflagellar transport 140 [32]. Dental pulp tissue is located in the closed and hard dentin wall, which determines its poor intramedullary circulation. Therefore, it is easy to cause edema of pulp tissue, then the pressure of pulp cavity increases which leading to the occurrence of pulpitis and finally pulp necrosis occur. As known, pulpitis can be caused by caries, trauma and chemical irritation from dental materials; pulpal inflammation ultimately activates the complement system leading to dental pulp tissue damage. Following complement activation, a complement protein is secreted that binds to its corresponding receptor and simultaneously chemotaxis-related immune cells migrate to the zone complement activation to drive immune function. Studies have reported that in addition to chemotactic immune cells, C5a induces DPSCs migration to the damaged area [33]. However, the role of C5a following the migration of DPSCs, in particular, whether C5a continues to display a role during the second stage of regeneration (the differentiation stage) has not been previously reported; therefore, the present study cultured DPSCs with different concentrations of C5a to determine the effects on DPSC differentiation.

In the present study, Cyagen Human Mesenchymal Stem Cell Growth Medium kits were used to culture the primary dental pulp cells and subsequently, stem cell-related marker expression was detected by flow cytometry. MSCA-1 was expressed by 5.6% of DPSCs, which was consistent with a previous report [21]. General high glucose DMEM with fetal bovine serum was used to culture DPSCs, which induced MSCA-1 expression in 1.1% of DPSCs, as determined by flow cytometry. The Cyagen Human Mesenchymal Stem Cell Growth Medium kits can aid with stem cell purification from the DPSC culture, and therefore, were the first choice for the DPSC experiments in the present study. We changed the culture medium every other day because of the cells proliferation during the long-term cell culture. Before conducting this study, we need to confirm the range of C5a concentration. The researchers cultured dental pulp cells with 1 nM C5a and detected the expression of DMP-1, and 1 nM C5a equivalent to 8.3 μg/ml [34], Other research reported that the nephrotic syndrome patients with relapse showed a higher serum C5a level (77.25 ng/ml) compared to remission status (36.93 ng/ml). The serum level of C5a in patients with nephrotic syndrome was higher (44.30 + 32.95 ng/ml) than in remission (22.31 + 14.62 ng /ml) [35]. The study on murine cortical tubular cells used 25 nM C5a (25 nM equal to 207.5 μg/ml) and detect the transforming growth factor- (TGF-β) expression [36]. Other study reported that they used 42 nM C5a (equals to 336 μg/ml) to culture mouse bone marrow cells [37]. At present, there are no relevant studies on the concentration of C5a in healthy pulp and inflammatory pulp. Therefore, we choose the concentration range from 50 to 400 ng/ml to show the difference in the differentiation of DPSC to odontoblasts. There was no significant difference in the expression levels of DSP mRNA and DSPP protein between the 50 ng/ml C5a group and the control group, which indicated that 50 ng/ml C5a did not influence DPSC differentiation, potentially because the concentration was too low. Therefore, 50 ng/ml C5a did not promote or inhibit DPSC differentiation compared with the control group. In the 100 and 200 ng/ml C5a groups, low mRNA and protein expression levels of the odontoblast cell markers DSP and DSPP were observed, which were 10 times lower compared with the control group. Therefore, concentrations of C5a between 100 and 200 ng/ml displayed a significant inhibitory effect on DPSC differentiation. Clinically, during deep caries or pulpitis, bacteria invade the pulp and induce complement secretion of C5a, which inhibits DPSC differentiation. It was hypothesized that failure to induce repair and regeneration in the dental pulp may be related to the concentration of C5a. The results of the present study indicated that 100 and 200 ng/ml C5a inhibited differentiation; however, this hypothesis requires further investigation. Inhibiting DPSC differentiation may be induced by certain concentrations of C5a, which may influence the self-repair process of dental pulp. However, the differentiation of DPSC displayed no apparent difference between the 300 ng/ml C5a group and the control group. The loss of inhibition observed at the high concentration of 300 ng/ml C5a implied that regeneration may occur at this concentration. Compared with the control group, a twofold increase in the expression of the odontoblast marker DSP was observed in the 400 ng/ml C5a group. Due to the mineralization inducer was used also in the control group. As the concentration of C5a increased, the effect of differentiation changed compared with the control group. The twofold high expression of DSP demonstrated the stronger differentiation promotion by 400 ng/ml C5a compared with the control group. Therefore, there is no toxic effect of C5a in 400 ng/ml C5a group. The present study did not evaluate the effect of C5a concentrations > 400 ng/ml on differentiation. Collectively, the results indicated that C5a displayed an effect on the proliferative activity and differneitiation capacity of DPSCs. The study of appropriate C5a concentration for pulp regeneration process can help to modified the existing traditional pulp treatment methods (such as root canal therapy) in future clinical diagnosis and treatment. Futhermore, the study may help to prevent the occurrence of pulpitis and protect the pulp activity in order to prolong the life of teeth in the mouth.

Previous studies have indicated that there is high C5a expression within 48 h and low C5a expression between 48 h and 7 days post-LTA and LPS stimulation of human dental pulp cells. Although the expression pattern in vitro cannot simulate the state of the body, the present study indicated that high C5a concentrations significantly promoted the differentiation of DPSCs into odontoblasts, while the lower concentration of C5a such as 100 and 200 ng/ml used in the present study displayed an obvious inhibitory effect. Therefore, in the clinical case of deep caries, once the complement response is activated, alterations to the levels of C5a may display a key role during the repair and regeneration of the pulp. During the immune process in the dental pulp, the concentration of C5a initially secreted low, then the expression of C5a gradually increased as the immune response progressed. As C5a expression increases, its combination with C5aR can lead to immune cascades that induce further inflammatory factor secretion and immune cell recruitment, which is adverse to pulp regeneration. The immue cascades did not protect the pulp and prevent the occurrence of pulpitis in some cases. Therefore, controlling the expression and inducing high expression of C5a to promote DPSC differentiation to induce dental pulp regeneration may serve as an effective therapeutic strategy.

## Conclusions

Collectively, the present study demonstrated for the first time that high concentrations of C5a promoted DPSC differentiation. By contrast, llower concentrations of C5a displayed clear inhibition. This study showed the differentiation-promoting characteristics of different concentrations of C5a.The role of C5a in the development of pulp immunity deserves further study. To the best of our knowledge, the results of the present study may provide a theoretical basis for further investigation into the use of C5a as a therapeutic for deep decay that approaches the pulp.

## Data Availability

The datasets used for the current study are available from the corresponding author on reasonable request.

## Abbreviations

C5a: Complement component 5a
DPSCs: Dental pulp mesenchymal stem cells
DSP: Dentin sialoprotein
MSCs: Mesenchymal stem cells
DSPP: Dentin sialophosphoprotein
CD271: Nerve growth factor receptor
MSCA-1: Mesenchymal stem cell antigen
CD56: Neural cell adhesion molecule
LTA: Lipoteichoic acid
LPS: Lipopolysaccharide
